# Craniofacial Radiographic Features in Amelogenesis Imperfecta: A Case-Control Study

**DOI:** 10.7759/cureus.96417

**Published:** 2025-11-09

**Authors:** Kawtar Chadli, Mustapha El Alloussi, Fatima Zaoui, Abdelali Halimi

**Affiliations:** 1 Orthodontics and Dentofacial Orthopedics, Faculty of Dental Medicine, Mohammed V University de Rabat, Rabat, MAR; 2 Center for Research in Health Sciences (CRESS), Faculty of Dentistry, International University of Rabat, Rabat, MAR

**Keywords:** amelogenesis imperfecta, cephalometry, craniofacial, open bite, skeletal malocclusion

## Abstract

Objectives

This study aimed to assess the craniofacial characteristics of individuals with amelogenesis imperfecta (AI) through cephalometric data analysis.

Methods

The sample comprised 19 patients (11 female, 8 male), with a mean age of 14.00 ± 4.61 years, diagnosed with AI, and 40 age- and sex-matched healthy controls with normal occlusion and balanced profiles. Twenty-three cephalometric variables were used for quantitative assessment. Differences were compared between groups using Student’s t-test (p < 0.05).

Results

Open-bite malocclusion was observed in 57.9% of individuals with AI. The vertical skeletal relationship showed increased anterior facial height, mandibular plane angle, and gonial angles compared to controls (p < 0.01). The skeletal sagittal relationship exhibited a Class II pattern with a retruded mandibular position (p < 0.01), while the maxillary and mandibular lengths showed no significant differences. Additionally, analysis across the four main AI subtypes revealed no significant differences in craniofacial morphology (p > 0.05).

Conclusions

Excessive vertical growth appears to be the primary cause of the high prevalence of anterior open bite (AOB) in AI patients. These findings suggest that AI-related enamel defects may indirectly influence skeletal growth patterns, warranting early orthodontic monitoring and specialized care.

## Introduction

Amelogenesis imperfecta (AI) is a rare genetic disorder that encompasses a heterogeneous group of conditions primarily affecting the dental enamel. AI may present as an isolated condition or as part of syndromes with other systemic features. Clinically, it manifests in both primary and permanent dentitions, causing discolored, fragile, and sensitive teeth, with functional and aesthetic consequences depending on the severity of the condition [1].

The global prevalence of AI varies significantly, ranging from 1/700 to 1/14,000 depending on the population studied, with an estimated global average of 0.5% [2]. To the best of our knowledge, no studies to date have investigated its prevalence in Moroccan or North African populations.

Diagnosing AI is complex due to its phenotypic diversity, which includes enamel defects such as hypoplasia and hypomineralization, often coexisting within the same individual or tooth. Diagnosis typically relies on family history, clinical observation, pedigree analysis, and radiographic imaging to assess enamel mineralization [1]. In addition to enamel abnormalities, AI is frequently associated with dental anomalies such as eruption disturbances, congenitally missing teeth, root and crown resorption, pulpal calcifications, and taurodontism [3]. Notably, studies have reported increased prevalence of anterior open bite (AOB) malocclusion with AI ranging from 24% to 60% [4,5]. Despite the growing body of research pointing to the frequent co-occurrence of these two disorders, the underlying etiopathogenic mechanisms linking AI and AOB are yet to be determined.

Several hypotheses have been proposed to explain this association. Early theories linked it to tongue interposition caused by dental sensitivity, thus attributing it to a dentoalveolar origin [4]. Others suggest that open bite is a feature of a specific form of AI, potentially reflecting the pleiotropic effects of AI gene mutations on craniofacial development and alveolar growth [5]. However, no definitive evidence supports a pleiotropic mechanism linking AI and skeletal dysplasia [6,7]. Others suggest a multifactorial etiology, involving interactions between modifying genetic effects and environmental factors [8,9].

This study aims to (1) compare the vertical and sagittal craniofacial characteristics of Moroccan AI patients with those of Moroccan individuals with normal occlusion, (2) investigate the relationship between AI and AOB malocclusions, and (3) identify any associations between specific AI phenotypes and dentofacial malocclusions.

## Materials and methods

Study design

This retrospective descriptive case-control study was conducted to analyze craniofacial characteristics of patients diagnosed with AI and a control group using cephalometric data.

Study setting and participants

This study was conducted in March 2025, based on data collected from radiographic records of AI patients treated between 2017 and 2024 at the Department of Orthodontics and Dentofacial Orthopedics, Mohammed V University, Rabat, Morocco. Fifty-nine Moroccan participants were included: 19 patients diagnosed with AI and 40 healthy controls. AI patients were selected based on confirmed clinical diagnosis and availability of lateral cephalometric and panoramic radiographs. Patients with clinical findings consistent with a syndromic condition were excluded. Controls were selected among individuals who consulted at the Department of Orthodontics and Dentofacial Orthopedics, Mohammed V University in Rabat. Selection criteria for controls included Class I skeletal and occlusal relationships, normal overbite, and well-balanced facial profiles. Controls were matched to AI cases at a 2:1 ratio based on age (±1 year), sex, and ethnicity to minimize confounding effects.

Ethical considerations

The study was approved by the Ethics Committee for Biomedical Research (CERB) under approval number 04/25 in February 2025. The purpose and procedures of the study were explained to all participants, and written informed consent was obtained from participants and/or their parents. The study was conducted in accordance with the Declaration of Helsinki.

Data sources and measurement

**Figure 1:**
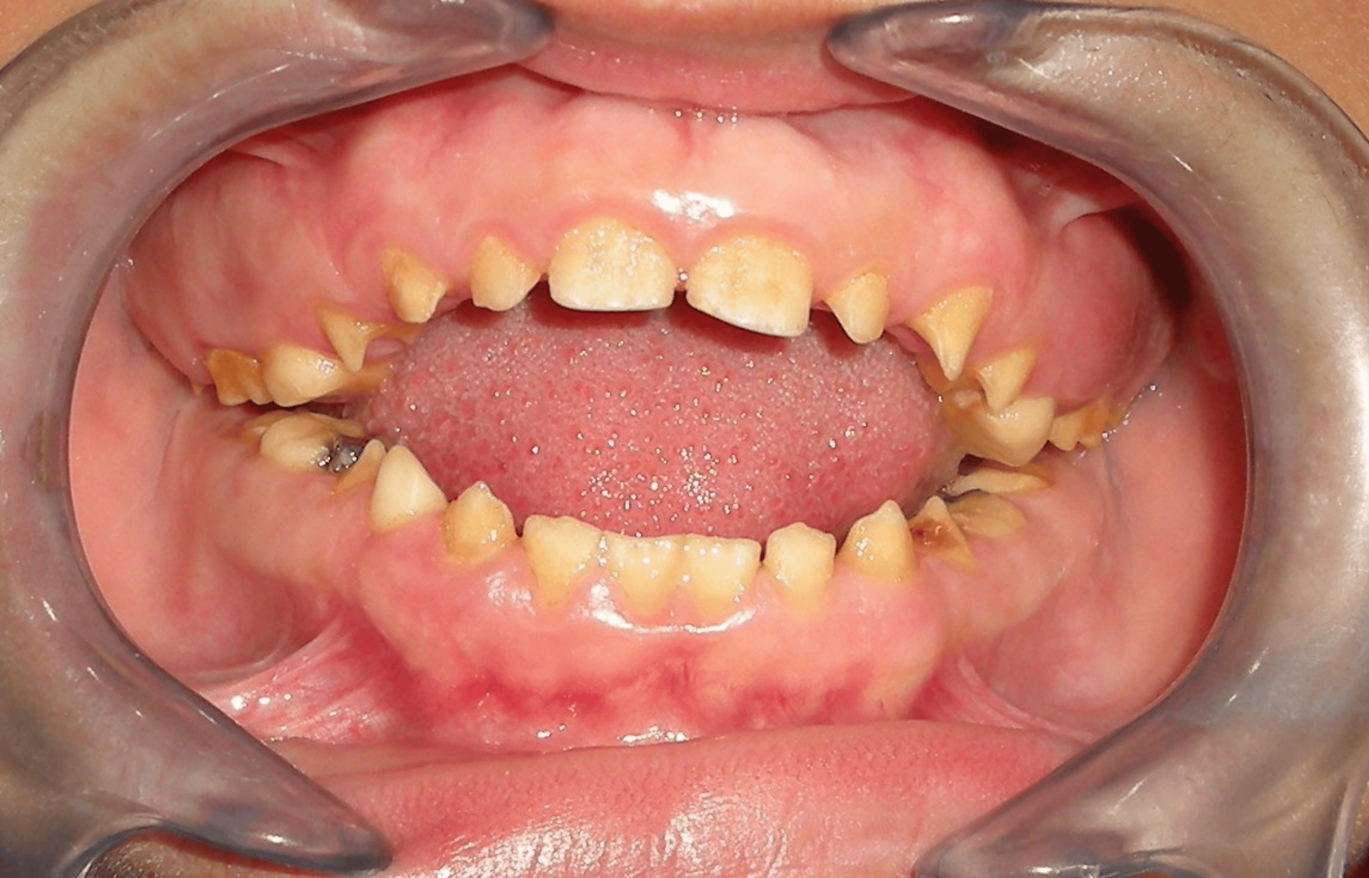
Intraoral photograph of a 14-year-old patient with type I hypoplastic amelogenesis imperfecta, illustrating enamel defects and skeletal anterior open-bite malocclusion.

**Figure 2:**
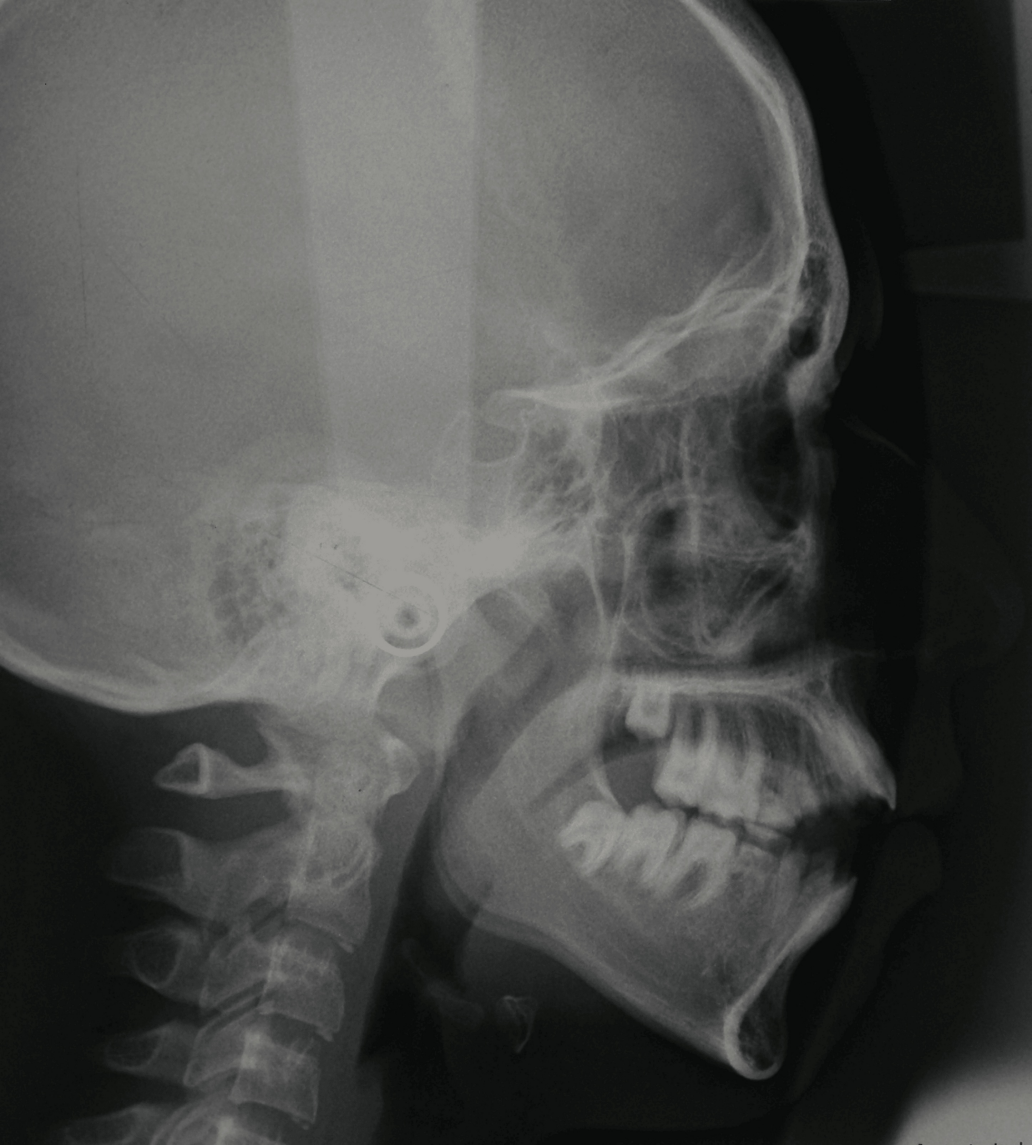
Lateral cephalometric radiograph of the patient shown in Figure 1, diagnosed with amelogenesis imperfecta and anterior open bite.

Cephalometric and digital panoramic radiographs were obtained under standardized conditions with the head in a natural position, stabilized by ear rods. The exposure settings for the radiographs were as follows: 80 kVp, 12 mA, and 0.63 seconds for cephalometric radiographs and 68 kVp, 8 mA, and 13.5 seconds for panoramic radiographs. An intraoral photograph (Figure 1) and a representative lateral cephalometric radiograph of an AI patient (Figure 2) are provided.

All cephalometric measurements were carried out by a single observer (C.K.) using AutoCAD LT 2025 software (Autodesk, San Francisco, CA, USA). The magnification was accounted for in the linear measurements, and dual images of bilateral anatomical landmarks were bisected. Definitions of the landmarks used in this study are provided in Table 1.

**Table 1:** Cephalometric landmarks used in this study for linear and angular measurements of Moroccan patients with amelogenesis imperfecta and matched controls.

Landmark name	Landmark abbreviation
Nasion	N
Sella turcica	S
Basion	Ba
Articulare	Ar
Condylion	Co
Orbitale	Or
Anatomical porion	Po
Posterior nasal spine	PNS
Anterior nasal spine	ANS
A point	A
B point	B
Pogonion	Pg
Gnathion	Gn
Menton	Me
Gonion	Go
Upper incisor edge	UIE
Upper incisor apex	UIA
Lower incisor edge	LIE
Lower incisor apex	LIA
Upper molar mesial cusp tip	U6M
Lower molar mesial cusp tip	L6M

Variables

**Figure 3:**
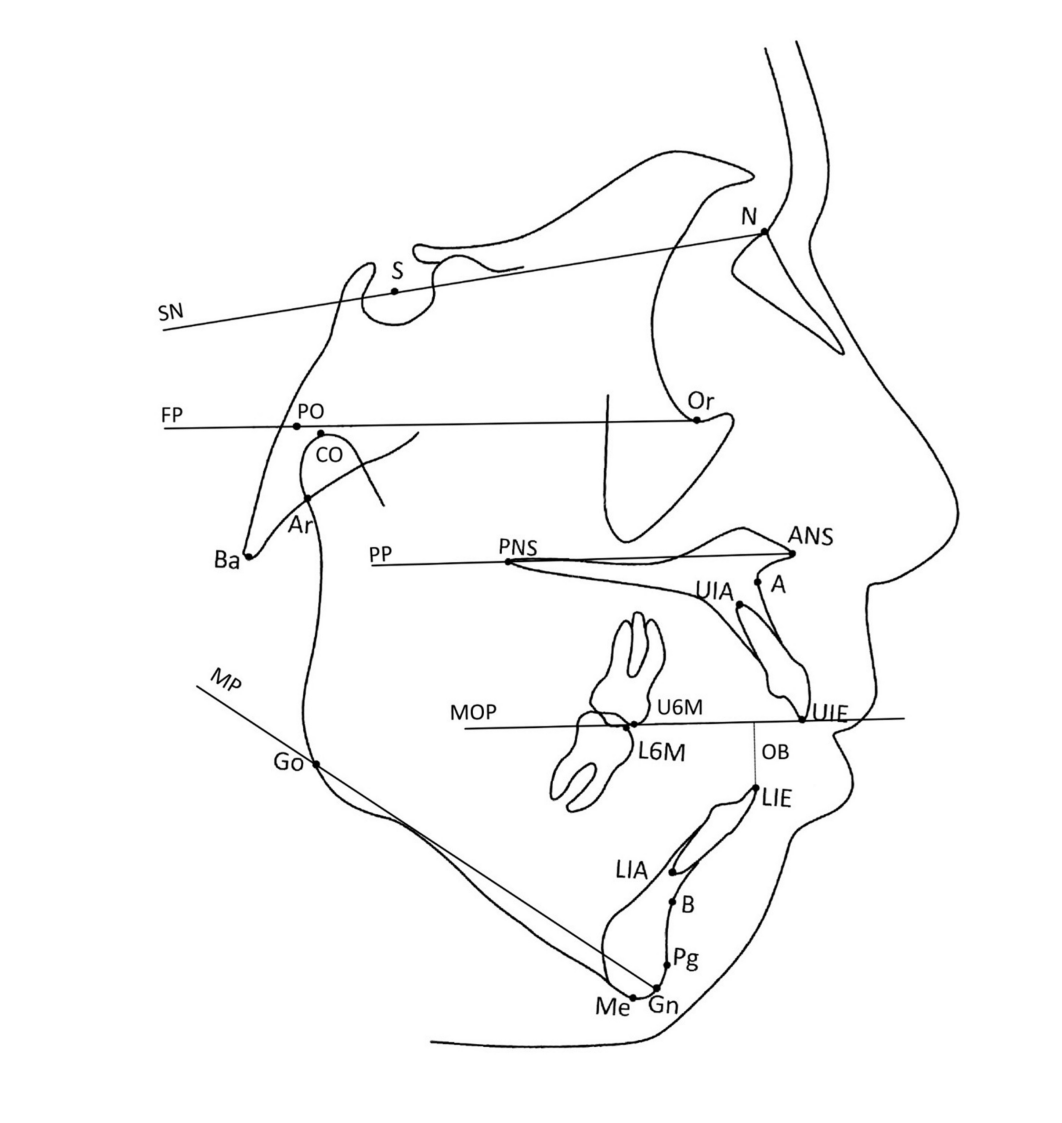
Representative cephalometric tracing with reference lines: SN plane, mandibular plane (MP), Frankfort plane (FP), palatal plane (PP), overbite (OB), maxillary occlusal plane (MOP) traced between the upper molar mesial cusp tip and upper incisor edge. Image Credit: Author's original creation.

The outcome variables were cephalometric parameters reflecting vertical and sagittal facial growth. Twenty-three cephalometric variables (12 linear and 11 angular) were used. Four primary measures were overbite, mandibular plane angle, gonial angle, and anterior facial height. The overbite parameter, referring to the vertical relationship of the incisors, was measured by incisor overlap perpendicular to the maxillary occlusal plane (Figure 3). Negative values indicated the presence of dental AOB malocclusion. Skeletal open bite was characterized by an increased gonial angle, a steep mandibular plane angle, and excessive lower anterior facial height. A positive diagnosis was defined by at least two of these measures being more than two Z-scores above the mean for age and gender matched controls. This method has been described in previous studies as a more accurate and objective approach for defining this phenotype [10].

AI subtype was considered a categorical variable. Individuals with AI were diagnosed by an international consortium based on clinical and radiographical findings and classified for data analysis according to Witkop's classification, which clinically distinguishes four primary groups: hypoplastic AI (Type I), hypomature AI (Type II), hypomineralized AI (Type III), and hypoplastic-hypomature AI with taurodontism (Type IV), which represents a combination of Types I and II [10,11].

Bias

Intra-observer reliability was assessed by repeating the analysis of 10 randomly selected radiographs at a one-week interval, with intraclass correlation coefficients (ICCs) confirming high reproducibility (ICC > 0.9) for cephalometric measurements.

Study size

All AI cases meeting the inclusion criteria were included. Sample size was not calculated due to the rarity of AI.

Statistical methods

Statistical analysis was performed using IBM SPSS Statistics for Windows, Version 30.0 (Released 2024; IBM Corp., Armonk, NY, USA). Cephalometric variables were treated as continuous. Normality and distribution of data were assessed using the Kolmogorov-Smirnov test. Z-scores for the four primary measures were computed using age- and gender-matched controls with significance set at Z ≥ 1.96. Differences between AI and control groups were analyzed using Student’s t-test for quantitative variables, with a significance threshold of p < 0.05. Subgroup analyses were conducted using ANOVA followed by Bonferroni’s post hoc test. Sexual dimorphism among AI cases was assessed using independent samples t-tests. There were no missing data in the final dataset.

## Results

Of the 27 AI cases initially reviewed, 6 were excluded due to incomplete radiographic records and 2 due to associated syndromic conditions. A total of 19 AI cases were retained for analysis. The demographic characteristics of the study cohort are summarized in Table 2. The mean age was comparable between the AI group (14.00 ± 4.61 years) and the matched control group (13.90 ± 4.60 years).

**Table 2:** Clinical characteristics of study participants with amelogenesis imperfecta and age-, sex-, and ethnicity-matched controls. Data were collected from the Department of Orthodontics, Mohammed V University, Rabat, Morocco, from 2017 to 2025 (N = 59).

Parameter	AI subjects	Controls
Mean age	14.00 ± 4.61	13.90 ± 4.60
Gender		
Female	11	22
Male	8	18
Total participants	19	40

The phenotype distribution of AI cases showed the highest proportions for hypoplastic AI (31.58%, n = 6) and hypomature AI (31.58%, n = 6). A lower proportion was observed for hypomineralized AI (21.05%, n = 4). Mixed types, including hypomaturation/hypoplasia with taurodontism, were diagnosed in 15.79% (n = 3) of cases.

Outcome data

**Figure 4:**
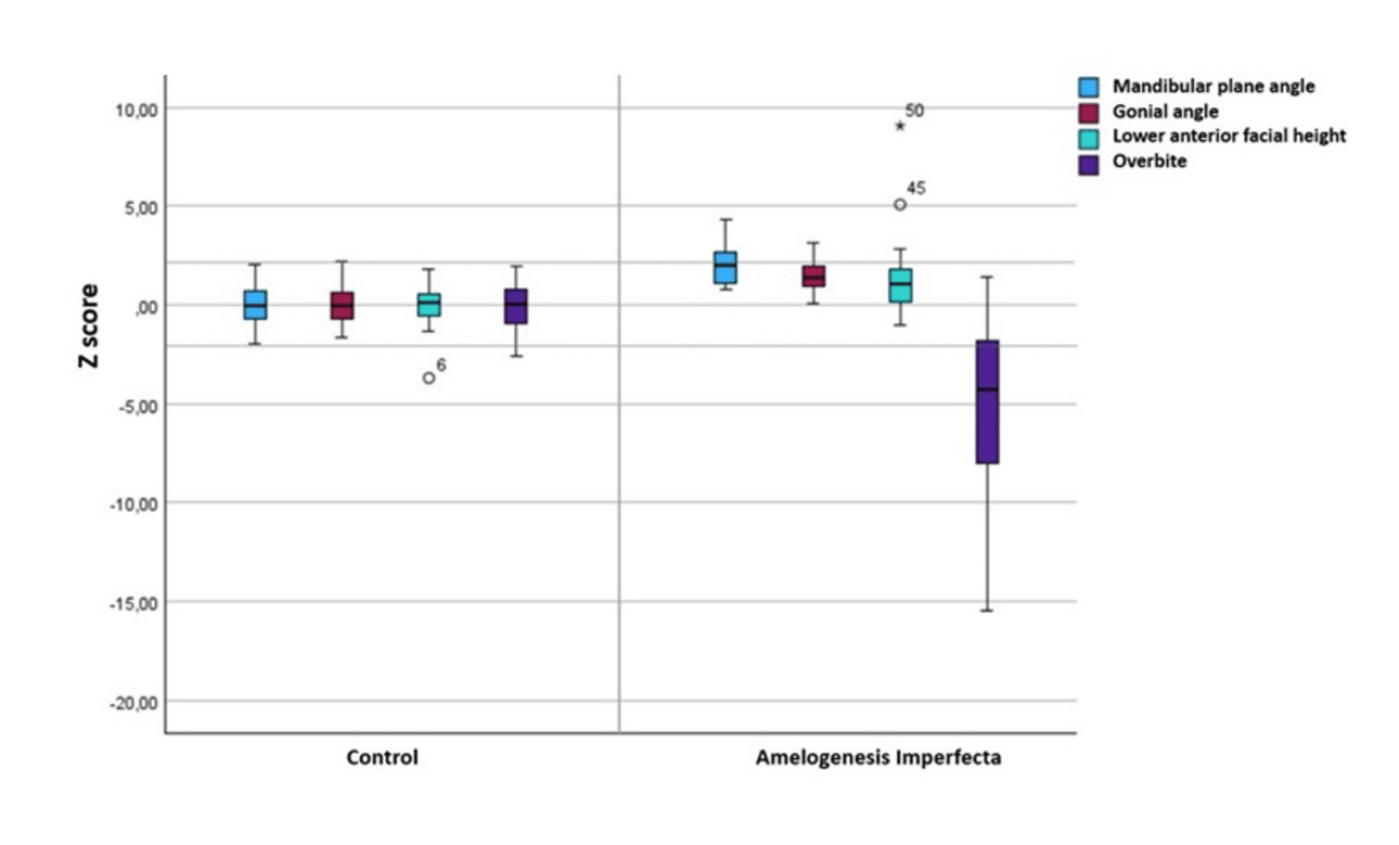
Distribution of Z-scores for cephalometric deviations from normal values in subjects with amelogenesis imperfecta. The dotted horizontal line represents two standard deviations from the mean Z-score. Lower anterior facial height (Me-ANS), mandibular plane angle (SN/MP), gonial angle (Me-Go-Ar), and overbite (OB).

The results of the cephalometric analysis are shown in Table 3. Eleven of the 19 patients with AI (57.9%) had negative overbite values, indicating open-bite malocclusion. For AI patients, the mean overbite measurement (-2.49 ± 4.52) was significantly more negative (p < 0.01) than the control group (2.50 ± 0.96). Out of the 19 cases analyzed, 5 cases (26.3%) exhibited at least two measures with Z-scores exceeding this threshold. The distribution of Z-scores is shown in Figure 4.

**Table 3:** Bivariate analysis (Student’s t-test) of cephalometric measurements comparing amelogenesis imperfecta cases and controls (N = 59). Values are expressed as mean ± SD. p < 0.05 was considered statistically significant. ns: not significant, t(df): Student’s t-test (Welch correction for unequal variances). *p < 0.05. **p < 0.01.

Variables		AI (mean ± SD)	Control (mean ± SD)	t(df)	p-value
Sagittal cephalometric measurements					
SNA (°)		78.21 ± 3.24	80.35 ± 3.40	-2.33 (37.1)	*
SNB (°)		74.26 ± 3.21	78.20 ± 3.24	-4.39 (35.8)	**
ANB (°)		3.95 ± 2.39	2.15 ± 1.58	2.99 (25.7)	**
N-S-Ba (°)		132.53 ± 4.75	131.68 ± 5.03	0.63 (37.4)	ns
CoA (mm)		79.11 ± 7.73	80.93 ± 5.04	-0.94 (25.5)	ns
CoGn (mm)		104.94 ± 12.82	106.17 ± 8.06	-0.38 (25.0)	ns
SN (mm)		66.45 ± 7.09	65.37 ± 4.02	0.62 (23.7)	ns
Vertical cephalometric measurements					
PP/MP (°)		36.00 ± 4.76	25.78 ± 5.09	7.53 (37.7)	**
SN/PP (°)		6.32 ± 2.89	6.65 ± 3.21	-0.40 (39.1)	ns
SN/MP (°)		42.00 ± 4.86	32.23 ± 4.77	7.26 (34.9)	**
FH/MP (°)		31.11 ± 4.91	25.05 ± 4.03	4.68 (29.9)	**
Me-Go-Ar (°)		136.47 ± 5.59	127.28 ± 6.21	5.69 (39.1)	**
Ar-Go (mm)		38.79 ± 4.75	41.92 ± 5.35	-2.27 (39.6)	*
N-Me (mm)		110.43 ± 18.30	110.60 ± 8.57	-0.04 (21.8)	ns
Me-ANS (mm)		72.77 ± 16.17	62.59 ± 7.03	2.63 (21.3)	**
N-ANS (mm)		48.15 ± 5.60	48.06 ± 4.25	0.06 (28.2)	ns
	Dental position and inclination				
UIE–UIA/PP (°)		110.89 ± 5.68	110.88 ± 6.99	0.01 (43.0)	ns
LIE–LIA/MP (°)		91.89 ± 9.63	95.20 ± 6.16	-1.37 (25.2)	ns
OB (mm)		-2.49 ± 4.52	2.50 ± 0.96	-4.76 (18.8)	**
UIE-PP (mm)		27.92 ± 3.61	27.98 ± 3.03	-0.06 (30.5)	ns
LIE-MP (mm)		35.35 ± 4.08	34.35 ± 3.03	0.95 (27.8)	ns
U6M-PP (mm)		22.01 ± 3.48	21.72 ± 2.49	0.33 (27.1)	ns
L6M-MP (mm)		26.96 ± 3.89	26.84 ± 2.84	0.12 (27.5)	ns

In the sagittal dimension, significant differences were observed in the skeletal sagittal relationship, involving both maxillary and mandibular positions for AI patients. Specifically, the SNB angle was significantly smaller (74.26 ± 3.21, p < 0.01), and the ANB angle was larger, indicating a tendency toward a Class II skeletal pattern. However, no significant differences were found in effective maxillary dimension (Co-A) or mandibular dimension (Co-Gn) between the groups. Additionally, there were no significant differences in anterior cranial base length (S-N) or cranial base angle (N-S-Ba).

Highly significant differences in the vertical skeletal relationship were observed in the AI group. These were characterized by a greater mandibular plane angle (SN/MP: 42.00 ± 4.86, p < 0.01) and confirmed by a larger intermaxillary angle (PP/MP) (36.00 ± 4.76, p < 0.01) compared to controls. AI patients exhibited more obtuse gonial angles (Me-Go-Ar) (p < 0.01), increased anterior facial height (Me-ANS) (72.77 ± 16.17, p < 0.01), and decreased lower posterior facial height (Ar-Go) (p < 0.05). However, no significant differences were observed in the maxillary inclination angle (SN/PP), upper facial height (N-ANS), or total facial height (N-Me), indicating that the vertical excess originated primarily in the craniofacial component of the lower face.

No significant differences were observed in dental relationships between AI patients and controls, including the inclination of the upper and lower incisors and the vertical position of the first molars and incisors (p > 0.05).

Subgroup analysis

No significant sexual dimorphism was observed in cephalometric variables within the AI group. Additionally, analysis by AI subtypes revealed no significant differences in craniofacial characteristics, suggesting no apparent association between subtype and craniofacial morphology in the studied sample, as shown in Table 4.

**Table 4:** Subgroup analysis of cephalometric variables by amelogenesis imperfecta subtype according to Witkop’s classification using one-way ANOVA followed by Bonferroni’s post hoc test (N = 19). Values are expressed as mean ± SD. p < 0.05 was considered statistically significant. Bonferroni’s post-hoc test was applied, but no significant differences were found among subtypes (all p > 0.05). ns: not significant. F: F-statistic from one-way analysis of variance (ANOVA). *p < 0.05. **p < 0.01.

Variables	Hypomineralization (N = 4; mean value ± SD)	Hypomature (N = 6; mean value ± SD)	Hypoplastic (N = 6; mean value ± SD)	Mixed type (N = 3, mean value ± SD)	F-value	p-value	
Sagittal cephalometric measurements							
SNA (°)	76.00 ± 2.44	78.50 ± 3.45	79.50 ± 3.56	78.00 ± 3.00	0.95	ns	
SNB (°)	72.25 ± 3.20	74.00 ± 3.34	76.00 ± 3.52	74.00 ± 1.00	1.16	ns	
ANB (°)	3.75 ± 2.06	4.50 ± 3.01	3.50 ± 2.34	4.00 ± 2.64	0.16	ns	
N-S-Ba (°)	134.75 ± 3.50	134.67 ± 1.21	130 ± 6.02	129.67 ± 6.39	1.79	ns	
CoA (mm)	84.38 ± 8.07	74.48 ± 7.79	81.20 ± 6.09	77.16 ± 7.50	1.74	ns	
CoGn (mm)	113.36 ± 11.64	96.62 ± 9.58	110.5 ± 11.20	99 .52 ± 15.86	2.44	ns	
SN (mm)	72.94 ± 9.53	63.18 ± 5.69	65.51 ± 5.80	66.20 ± 5.63	1.79	ns	
Vertical cephalometric measurements							
PP/MP (°)	33.50 ± 4.43	36.83 ± 5.41	38.33 ± 4.59	33 ± 2.00	1.39	ns	
SN/PP (°)	8.50 ± 0.57	5.33 ± 3.07	6.83 ± 3.43	4.33 ± 1.52	1.72	ns	
SN/MP (°)	41.75 ± 4.19	41.83 ± 4.35	44.67 ± 5.82	37.33 ± 0.57	1.71	ns	
FH/MP (°)	28.75 ± 5.56	30.67 ± 4.71	34.00 ± 4.94	29.33 ± 3.51	1.19	ns	
Me-Go-Ar (°)	136.00 ± 4.54	136.83 ± 6.67	139.00 ± 4.98	131.33 ± 4.16	1.35	ns	
Ar-Go (mm)	43.14 ± 3.54	36.42 ± 1.98	37.74 ± 5.24	39.78 ± 6.80	2.07	ns	
N-Me (mm)	122.12 ± 8.69	100.86 ± 20.59	111.44 ± 18.80	111.94 ± 20.05	1.13	ns	
Me-ANS (mm)	69.44 ± 6.64	70.68 ± 14.02	80.26 ± 23.07	66.38 ± 14.08	0.63	ns	
N-ANS (mm)	53.32 ± 4.16	44.23 ± 6.00	49.38 ± 2.87	46.64 ± 6.15	3.08	ns	
Dental position and inclination							
UIE–UIA/PP (°)	109.25 ± 10.72	112.00 ± 3.68	110.67 ± 4.85	111.33 ± 3.78	0.17	ns	
LIE–LIA/MP (°)	92.25 ± 10.21	93.50 ± 10.05	87.67 ± 9.04	96.67 ± 11.15	0.65	ns	
OB (mm)	-2.34 ± 4.95	-1.25 ± 3.37	-2.26 ± 4.32	-5.58 ± 7.24	0.58	ns	
UIE-PP (mm)	27.20 ± 4.90	27.87 ± 1.40	29.51 ± 3.81	25.78 ± 4.96	0.76	ns	
LIE-MP (mm)	37.96 ± 2.84	33.36 ± 2.94	36.72 ± 4.21	33.12 ± 5.74	1.74	ns	
U6M-PP (mm)	23.92 ± 2.90	19.85 ± 1.63	23.13 ± 2.90	21.53 ± 6.54	1.53	ns	
L6M-MP (mm)	29.56 ± 4.67	25.00 ± 1.38	26.72 ± 1.28	27.84 ± 8.23	1.21	ns	

## Discussion

AI represents a diverse spectrum of clinical presentations rooted in genetic variability. Numerous studies have highlighted the association between AOB and AIB. A recent systematic review identified open bite as the most observed malocclusion, showing high prevalence across subtypes. It occurs in 46.8% of hypoplastic and hypomature AI cases and 45% of the hypomineralized AI phenotype, without a definitive link to any specific subtype [7].

This malocclusion, though clinically diagnosed, has been assessed through cephalometric analyses to evaluate underlying morphological factors. Thus, AOB malocclusion has been classified as either a dental open bite or skeletal open bite [12]. This study assessed lower anterior facial height (Me-ANS), mandibular plane angle (SN/MP), and gonial angle (Me-Go-Ar) as primary markers of the skeletal origin of AOB.

Additionally, eruption patterns and incisor inclination were analyzed to assess severity and occurrence in AI patients. The results confirm that open-bite malocclusion in AI cases is primarily of skeletal origin, with vertical malocclusion as the dominant feature regardless of negative overbite. Minimal differences in alveolar heights suggest a less-pronounced compensatory mechanism.

The observed craniofacial cephalometric characteristics are consistent with existing literature, which reports a higher prevalence of skeletal open bite, a long-face growth pattern, and increased mandibular plane inclination relative to the skull base [7]. Rowley et al. [6] reported vertical dysgnathia in 44% of AI patients and AOB in 24% associated with a severe discrepancy in the vertical relationship of the jaws, while Ravassipour et al. [11] found that 42% of Caucasian AI individuals exhibited dental and/or skeletal open-bite malocclusion.

Open-bite malocclusion has been reported in the Turkish population. Becerik et al. [13] observed AOBs associated with autosomal-recessive AI due to FAM83H mutations in 5/10 cases studied. In contrast, Oz et al. [14] found that only 35% of the 20 patients had AOB malocclusion. Sagittal discrepancies were also described by Wright et al. [15], who highlighted the association of Class III malocclusions with AI. Möhlhenrich et al. [16] noted an equal distribution between Class II and Class III malocclusions, increased overjet, and vertical discrepancies, with 36.8% presenting an open bite.

Our study sample showed no association with Class III malocclusion but exhibited a sagittal Class II tendency. The significant differences observed in sagittal and vertical dimensions may be attributed to ethnic diversity. Clinically, it is important to consider the cephalometric norms for the Moroccan population, as established by Ousehal et al. [17]. Their findings, consistent with our control group observations, showed greater skeletal sagittal discrepancies, a Class II tendency, retruded maxillary, and mandibular bases.

Persson and Sundell [5] observed more pronounced skeletal defects in males than in females, attributing it to prolonged mandibular growth, which limits dentoalveolar compensation. In contrast, our results showed no significant gender differences, consistent with the systematic review conclusion that sexual dimorphism in linear and angular skeletal dimensions was absent [7]. Moreover, our findings suggest that the lack of dentoalveolar compensation is most likely linked to atypical tongue swallowing and low tongue posture at rest, emphasizing the multifactorial etiology of open-bite malocclusion. Individuals with a normal overbite may display subtle differences in anterior and posterior tooth eruption [18]. The correlations between alveolar heights and vertical skeletal patterns, particularly in the anterior regions, suggest a compensatory mechanism in vertical facial development, emphasizing the complex interaction between alveolar and skeletal growth [19]. Additionally, no significant differences were found in the inclination of the upper and lower anterior teeth, consistent with the findings of Möhlhenrich et al. [16].

Our findings align with prior studies, including those by Rowley et al. [6], Ravassipour et al. [11], and Möhlhenrich et al. [16], which similarly reported a high prevalence of AOB in AI patients associated with vertical dysgnathia and increased vertical facial dimensions. However, unlike some studies that described Class III sagittal patterns, our sample demonstrated a Class II skeletal tendency. Overall, our results support the literature while providing region-specific data.

Excessive vertical growth appears to be the primary cause of the high prevalence of AOB in AI patients. The association between AI and vertical skeletal discrepancies is likely genetic, though not fully explored. Over 70 causal genes for AI have been identified to date, with newly reported variants implicated in enamel development. Several of these genes are associated with both syndromic and non-syndromic rare diseases, positioning AI as a potential manifestation of a broader genetic expression profile [20].

Among the genes most frequently involved in AI, AMELX and ENAM are most frequently associated with open-bite malocclusion [11]. This suggests a potential role in lower facial height anomalies despite these genes being primarily expressed in teeth. However, this association between OB and ENAM has not been systematically found [7]. Facial growth and craniofacial morphology are influenced by both genetic and epigenetic factors, which contribute to the variability in malocclusion expression through complex gene-environment interactions [21]. Identifying shared mechanisms with AI could enhance treatment strategies for co-occurring disorders.

This study has several limitations. First, the sample size was small due to the rarity of AI, which may limit the statistical power of subgroup analyses. Second, both groups were recruited from an orthodontic specialty clinic, which may introduce referral and selection bias, as individuals with more severe malocclusions are more likely to be referred for specialist care. Third, the current AI classification into four main groups relies on clinical assessment and the exclusion of other dental anomalies, as genetic investigation remains limited to a few cases. The absence of genetic testing precluded analysis of genotype-phenotype correlations. To our knowledge, this is the first cephalometric study of Moroccan patients with AI, providing insight into population-specific craniofacial characteristics and vertical skeletal patterns associated with AOB.

Early intervention in AI patients should focus on restorative care and myofunctional therapy. While managing AOB is crucial, controlling the inherited skeletal pattern remains challenging due to limited evidence on treatment effectiveness and long-term stability [22]. To date, very little data are available concerning interceptive treatment in children with AI. Managing AI patients requires addressing both functional and psychosocial aspects, as they experience higher social avoidance and a reduced quality of life [23]. Given the restorative and orthodontic challenges posed by AI, a multidisciplinary approach, with full-mouth rehabilitation and, in some cases, orthognathic surgery, is essential to achieve optimal functional and aesthetic outcomes [24].

Key findings

A high prevalence of skeletal AOB (57.9%) was observed in AI patients, suggesting a vertical growth pattern beyond enamel defects. AI subjects exhibited increased anterior facial height, mandibular plane angle, and gonial angle compared with controls. Moroccan AI patients demonstrated a Class II skeletal tendency with mandibular retrusion. No significant craniofacial differences were found among AI subtypes.

## Conclusions

Open-bite malocclusion was observed in 57.9% of patients with AI, along with a Class II skeletal tendency. Excessive vertical growth appears to be associated with the high prevalence of open bite in these patients. These craniofacial characteristics underscore the need for early orthodontic evaluation to monitor vertical growth and developing malocclusion. Given the enamel fragility and restorative challenges in AI, coordinated care between pediatric dentists and orthodontists is recommended, with early interceptive orthodontics and timely restorative treatment to minimize functional and esthetic complications. These findings should be viewed in light of the single-center sample.

## References

[1] Crawford PJ, Aldred M, Bloch-Zupan A (2007). Amelogenesis imperfecta. Orphanet J Rare Dis.

[2] Gadhia K, McDonald S, Arkutu N, Malik K (2012). Amelogenesis imperfecta: an introduction. Br Dent J.

[3] Kammoun R, Ghoul S, Chaabani I, Ben Salem K, Ben Alaya T (2024). Dental and jawbone abnormalities linked to amelogenesis imperfecta: a retrospective and analytic study comparing panoramic radiographs. Spec Care Dentist.

[4] Witkop CJ (1957). Hereditary defects in enamel and dentin. Acta Genet Stat Med.

[5] Persson M, Sundell S (1982). Facial morphology and open bite deformity in amelogenesis imperfecta: a roentgenocephalometric study. Acta Odontol Scand.

[6] Rowley R, Hill FJ, Winter GB (1982). An investigation of the association between anterior open-bite and amelogenesis imperfecta. Am J Orthod.

[7] Broutin A, Bidi-Lebihan AK, Canceill T, Vaysse F, Bloch-Zupan A, Bailleul-Forestier I, Noirrit-Esclassan E (2023). Association between malocclusions and amelogenesis imperfecta genotype and phenotype: a systematic review. Int Orthod.

[8] Bäckman B, Adolfsson U (1994). Craniofacial structure related to inheritance pattern in amelogenesis imperfecta. Am J Orthod Dentofacial Orthop.

[9] Cartwright AR, Kula K, Wright TJ (1999). Craniofacial features associated with amelogenesis imperfecta. J Craniofacial Genet Dev Biology.

[10] Witkop CJ Jr (1988). Amelogenesis imperfecta, dentinogenesis imperfecta and dentin dysplasia revisited: problems in classification. J Oral Pathol.

[11] Ravassipour DB, Powell CM, Phillips CL, Hart PS, Hart TC, Boyd C, Wright JT (2005). Variation in dental and skeletal open bite malocclusion in humans with amelogenesis imperfecta. Arch Oral Biol.

[12] Cangialosi TJ (1984). Skeletal morphologic features of anterior open bite. Am J Orthod.

[13] Becerik S, Cogulu D, Emingil G, Han T, Hart PS, Hart TC (2009). Exclusion of candidate genes in seven Turkish families with autosomal recessive amelogenesis imperfecta. Am J Med Genet A.

[14] Oz U, Altug AT, Arikan V, Orhan K (2010). Radiographic evaluation of craniofacial structures associated with amelogenesis imperfecta in a Turkish population: a controlled trial study. Oral Radiol.

[15] Wright JT, Frazier-Bowers S, Simmons D (2009). Phenotypic variation in FAM83H-associated amelogenesis imperfecta. J Dent Res.

[16] Möhlhenrich SC, Chhatwani S, Schmidt P (2024). Orthodontic findings and treatment need in patients with amelogenesis imperfecta: a descriptive analysis. Head Face Med.

[17] Ousehal L, Lazrak L, Chafii A (2012). Cephalometric norms for a Moroccan population. Int Orthod.

[18] Dung DJ, Smith RJ (1988). Cephalometric and clinical diagnoses of open bite tendency. Am J Orthod Dentofacial Orthop.

[19] Abdelali H, Benyahia H, Abouqal R, Azaroual MF, Zaoui F (2012). Associations between alveolar heights and vertical skeletal pattern in Moroccan adults: a cephalometric study of 127 clinical cases. Int Orthod.

[20] Bloch-Zupan A, Rey T, Jimenez-Armijo A (2023). Amelogenesis imperfecta: next-generation sequencing sheds light on Witkop's classification. Front Physiol.

[21] Moreno Uribe LM, Miller SF (2015). Genetics of the dentofacial variation in human malocclusion. Orthod Craniofac Res.

[22] Lentini-Oliveira DA, Carvalho FR, Rodrigues CG, Ye Q, Prado LB, Prado GF, Hu R (2014). Orthodontic and orthopaedic treatment for anterior open bite in children. Cochrane Database Syst Rev.

[23] Coffield KD, Phillips C, Brady M, Roberts MW, Strauss RP, Wright JT (2005). The psychosocial impact of developmental dental defects in people with hereditary amelogenesis imperfecta. J Am Dent Assoc.

[24] Hoppenreijs TJ, Voorsmit RA, Freihofer HP (1998). Open bite deformity in amelogenesis imperfecta. Part 1: an analysis of contributory factors and implications for treatment. J Craniomaxillofac Surg.

